# Risk factors and predictive indicators of rupture in cerebral aneurysms

**DOI:** 10.3389/fphys.2024.1454016

**Published:** 2024-09-05

**Authors:** Xiguang Wang, Xu Huang

**Affiliations:** Department of Research & Development Management, Shanghai Aohua Photoelectricity Endoscope Co., Ltd., Shanghai, China

**Keywords:** cerebral aneurysm, morphology, hemodynamics, imaging modalities, blood biomarkers, artificial intelligence, fluid-structure interaction

## Abstract

Cerebral aneurysms are abnormal dilations of blood vessels in the brain that have the potential to rupture, leading to subarachnoid hemorrhage and other serious complications. Early detection and prediction of aneurysm rupture are crucial for effective management and prevention of rupture-related morbidities and mortalities. This review aims to summarize the current knowledge on risk factors and predictive indicators of rupture in cerebral aneurysms. Morphological characteristics such as aneurysm size, shape, and location, as well as hemodynamic factors including blood flow patterns and wall shear stress, have been identified as important factors influencing aneurysm stability and rupture risk. In addition to these traditional factors, emerging evidence suggests that biological and genetic factors, such as inflammation, extracellular matrix remodeling, and genetic polymorphisms, may also play significant roles in aneurysm rupture. Furthermore, advancements in computational fluid dynamics and machine learning algorithms have enabled the development of novel predictive models for rupture risk assessment. However, challenges remain in accurately predicting aneurysm rupture, and further research is needed to validate these predictors and integrate them into clinical practice. By elucidating and identifying the various risk factors and predictive indicators associated with aneurysm rupture, we can enhance personalized risk assessment and optimize treatment strategies for patients with cerebral aneurysms.

## Introduction

Cerebral aneurysms, pathological dilatations of the cerebral artery walls, represent a substantial health risk due to their potential for rupture, which often show localized expansions of the arterial segment, typically occurring at the bifurcations of major arteries at the base of the brain, known as the Circle of Willis (Li et al., 2023; Thompson et al., 2015; Etminan and Rinkel, 2016; Kim D. W. et al., 2019; Brisman et al., 2006; Kayembe et al., 1984; Chalouhi et al., 2013; Diagbouga et al., 2018). Currently, cerebral aneurysms have been significantly affected patients’ physical and mental conditions, which shows adverse impacts on public health concern due to their potential rupture, even leading to high mortality and disability rates (Hackenberg et al., 2020; Yang et al., 2021; Findlay and Deagle, 1998; Ajiboye et al., 2015; Wiebers et al., 1992). When rupture of cerebral aneurysms occurs, it leads to subarachnoid hemorrhage, a catastrophic event often resulting in serious conditions such as hydrocephalus, cerebral vasospasm, rebleeding, or cerebral infarction. Each of these complications brings its own devastating impact, from neurological deficits and cognitive impairments to the risk of permanent disability or death, thereby underscoring the high stakes involved in the management of cerebral aneurysms (Macdonald and Schweizer, 2017; Hop et al., 1997; Ingall et al., 2000; Tawk et al., 2021; Petridis et al., 2017; Steiner et al., 2013; Grasso et al., 2017; Biller et al., 1988).

The likelihood of cerebral aneurysm rupture is influenced by a complex interplay of nonmodifiable and modifiable factors. Nonmodifiable factors include age, gender, and genetic predisposition. For example, there is a higher prevalence of aneurysms in older individuals, females, and those with a familial history of aneurysms. Conversely, modifiable factors encompass lifestyle and health conditions such as hypertension, smoking, and alcohol consumption. Hypertension, for instance, can exacerbate the hemodynamic stress exerted on the arterial walls, while smoking and excessive alcohol consumption contribute to arterial damage and inflammation, thereby predisposing the individual to aneurysm formation and growth (Chalouhi et al., 2013; Kleinloog et al., 2018; Jin et al., 2019; Munarriz et al., 2016; Texakalidis et al., 2019; Lindner et al., 2010).

The risk of aneurysm rupture varies among individuals, and early identification of those at high risk is crucial for timely intervention and prevention of catastrophic outcomes. Cerebral aneurysms can affect individuals of any age, gender, and ethnicity, although certain demographic and clinical factors are known to increase the risk of aneurysm formation and rupture. These risk factors include advanced age, female gender, family history of aneurysms, and certain medical conditions such as hypertension and arteriosclerosis (Rinkel et al., 1998; Backes et al., 2016; Korja et al., 2014; Cho et al., 2020). Additionally, the location and morphology of the aneurysm, as well as hemodynamics acting on the vessel wall, play critical roles in determining the risk of rupture (Lall et al., 2009; Jeong et al., 2009; Signorelli et al., 2018; Waqas et al., 2020). The vessel wall at these dilated segments, often referred to as the aneurysmal dome, is characterized by the loss of elastic and muscular components, rendering it prone to rupture under hemodynamic stress (Chalouhi et al., 2013; Texakalidis et al., 2019; Signorelli et al., 2018; Sadasivan et al., 2013; Duan et al., 2016; Wang et al., 2023; Siasos et al., 2015).

In recent years, an evolving body of research has begun to pivot towards a more comprehensive understanding of the role of hemodynamic forces in the initiation, growth, and rupture of cerebral aneurysms. Hemodynamics, essentially the study of blood flow and the forces it exerts on vessel walls, is now being increasingly recognized as a critical player in the physiopathology of intracranial aneurysms (Signorelli et al., 2018; Staarmann et al., 2019). A large array of studies has attempted to elucidate the specific hemodynamic factors that contribute to aneurysm pathophysiology. Wall shear stress (WSS), for instance, is a tangential force that blood flow applies to the vessel wall, and abnormal WSS has been implicated in the development, progression, and rupture of aneurysms. A number of these studies suggest that high WSS can induce endothelial dysfunction and inflammatory responses, which contribute to aneurysm initiation, while low WSS can cause direct mechanical damage, contributing to aneurysm growth and rupture (Staarmann et al., 2019; Zhang et al., 2016; Penn et al., 2011; Meng et al., 2014). In addition to above indexes, other hemodynamic parameters such as pressure, flow velocity, and flow patterns have also been investigated for their role in aneurysm behavior (Jirjees et al., 2020; Xiang et al., 2014). For example, studies using computational fluid dynamics have modeled aneurysmal blood flow to study the complex interactions between these hemodynamic parameters and aneurysm morphology (Kleinstreuer et al., 2007; Nazri et al., 2020).

Meanwhile, there is an increasing focus on identifying predictive markers that can improve the precision of risk assessment for cerebral aneurysm rupture. These markers encompass biomolecules that mirror the biological processes that contribute to aneurysm formation and growth, such as markers of inflammation, indicators of extracellular matrix remodeling, and genetic variants (Hussain et al., 2015; Husseini and Laskowitz, 2010; Jordan and Nyquist, 2010). Advances in imaging technology, computational fluid dynamics, and machine learning algorithms have facilitated the development of innovative predictive models for assessing the risk of aneurysm rupture (Diab et al., 2023; Hosny et al., 2018; Chen et al., 2022).

Despite the progress made in understanding the risk factors and predictive indicators of rupture in cerebral aneurysms, challenges remain in accurately predicting which aneurysms will rupture. Current risk assessment primarily relies on aneurysm size, with larger aneurysms often believed to have a higher propensity for rupture. Other criteria include aneurysm location, morphology, and patient clinical history, which are all believed to have varying degrees of influence on rupture risk.

The purpose of this review is to delve into the current knowledge regarding the risk factors and predictive indicators that are associated with the rupture of cerebral aneurysms. The review underscores the significance of a holistic approach to risk assessment, taking into account a wide array of factors. Additionally, it addresses the shortcomings of the current risk assessment model, which primarily relies on aneurysm size. The review emphasizes the necessity of adopting a more multifaceted strategy that encompasses a comprehensive range of mechanical, biological, and hemodynamic factors to enhance the accuracy of predicting aneurysm rupture. By gaining a deeper understanding of the factors that contribute to aneurysm rupture, we can improve personalized risk assessment and optimize treatment strategies for patients with cerebral aneurysms, ultimately reducing the incidence of rupture-related complications and improving patient outcomes.

## Clinical perspective

**FIGURE 1:**
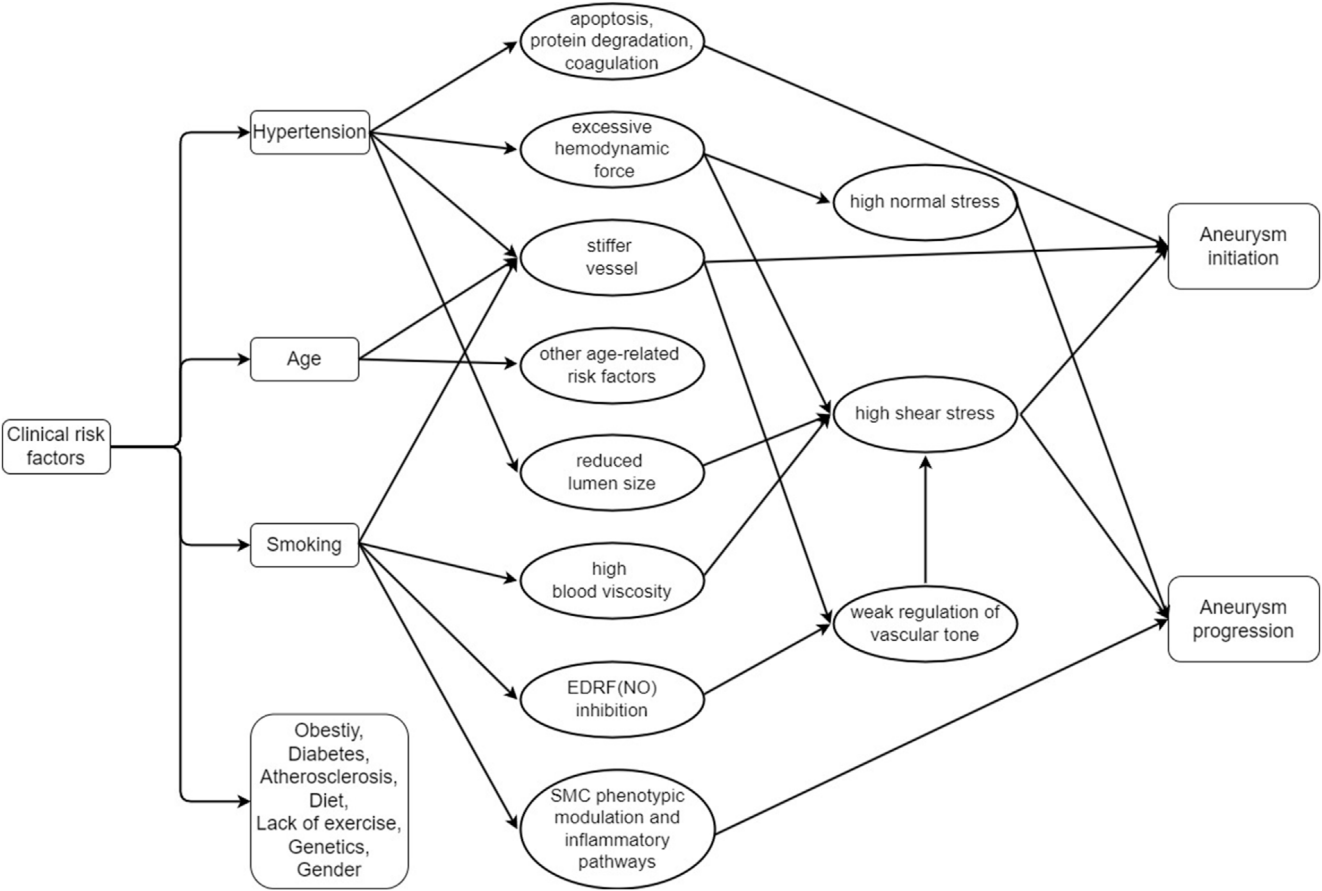
Relationship between the clinical risk factors and the initiation and progression of the cerebral aneurysm (EDRF: Endothelium-derived relaxation factor, NO: Nitric oxide, SMC: Smooth muscle cells).

The clinical perspective of rupture on cerebral aneurysms encompasses a comprehensive understanding of the risk factors, management strategies, and outcomes associated with this condition (Zhu et al., 2020). Herein, an overview of the clinical aspects of cerebral aneurysms rupture is shown, which highlights the importance of timely intervention and the potential for improved patient outcomes through early diagnosis and appropriate treatment, as shown in Figure 1.

To our knowledge, several risk factors including the age, gender, family history of aneurysms, hypertension, and arteriosclerosis are the distinguished information as the primarily routine reference from the clinicians (Kleinloog et al., 2018). Age is a nonmodifiable risk factor, with older individuals presenting a higher risk of aneurysm rupture due to the general degradation of vascular health over time (Brinjikji et al., 2016; Wermer et al., 2007). Sex is another factor, with females generally demonstrating a higher prevalence and rupture risk of intracranial aneurysms, potentially due to hormonal influences (Brinjikji et al., 2016; Turan et al., 2016). A patient’s clinical history, particularly a history of subarachnoid hemorrhage or familial history of aneurysms, significantly increases the rupture risk (Lee et al., 2021; Brown and Broderick, 2014; Rinkel, 2008).

The clinical presentation of cerebral aneurysm rupture could vary widely considering the physical diversity, but it often occurs suddenly with severe headache, neck stiffness, nauses, and vomiting (Etminan and Rinkel, 2016; Sadasivan et al., 2013; Wardlaw and White, 2000). Meanwhile, aneurysm with other symptoms may lead to altered consciousness, seizures, and focal neurological deficits. Ruptured aneurysms can occur subarachnoid hemorrhage, which can cause significant disability or death if not treated promptly.

The management of cerebral aneurysm involves a multidisciplinary approach, including the imaging, medical, and surgical interventions (Etminan and Rinkel, 2016; Brown and Broderick, 2014; Burkhardt et al., 2017). Imaging techniques play an important role in the diagnosis and monitoring of cerebral aneurysm. Medical management may include lifestyle modifications, blood pressure control, and the use of antiplatelet or anticoagulant medications. Surgical interventions including endovascular coiling or surgical clipping, is often required to prevent rupture and restore blood flow.

The outcomes of cerebral aneurysms rupture depend on various factors, including the time of diagnosis, the size and location of the aneurysm, the presence of complications, and the effectiveness of treatment (Kleinloog et al., 2018; Brinjikji et al., 2016; Ma et al., 2019). Early diagnosis and timely intervention are essential for improving outcomes and reducing the risk of disability or death. However, even with appropriate treatment, there is still risks of recurrence and the potential for long-term complications, including rebleeding and aneurysm-related stroke.

The clinical perspective on cerebral aneurysm rupture emphasizes the importance of early detection, timely intervention, and multidisciplinary management. By understanding the risk factors, clinical presentation, and management strategies, healthcare professionals can optimize patient care and improve outcomes of treatment for patients. However, the treatment of cerebral aneurysms is complex, and further research should concentrate on developing more effective prevention and treatment strategies.

## Morphological risk factors

**FIGURE 2:**
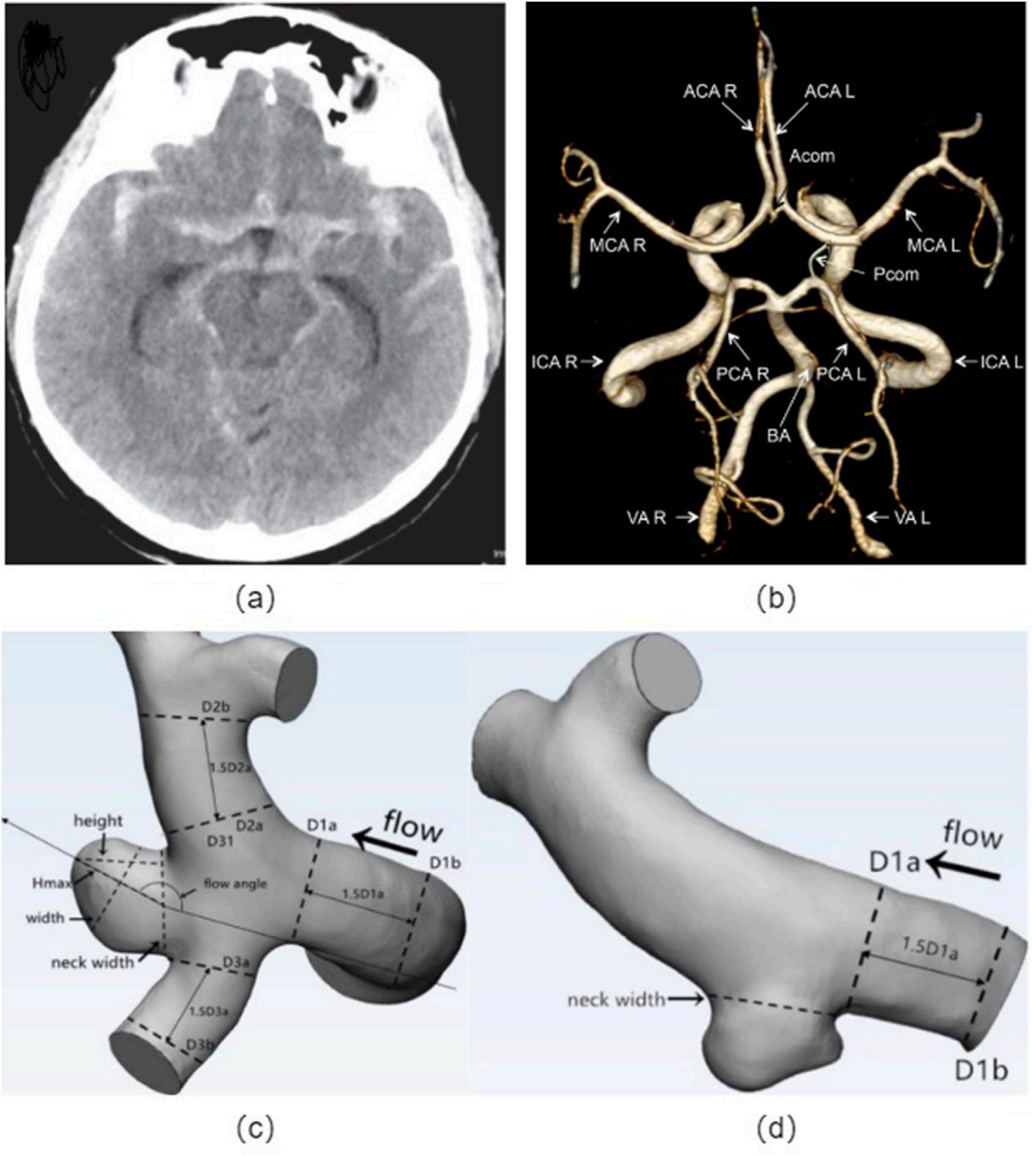
Cerebral artery morphology: **(A)** Non-contrast head CT showing subarachnoid hemorrhage from the left posterior communicating artery aneurysm; **(B)** Angiographic image of a human circle of Willis, indicated are: vertebral arteries (VA), right (R) and left (L), basilar artery (BA), internal carotid arteries (ICA R, ICA L), posterior cerebral arteries (PCA R, PCA L), anterior cerebral arteries (ACA R, ACA L), middle cerebral arteries (MCA R, MCA L), posterior communicating artery (Pcom) and anterior communicating artery (Acom); Definitions of morphological parameters in the bifurcation aneurysm **(C)** and sidewall aneurysm **(D)**. H_max_: the maximum distance of the dome from the centroid of the neck plane, Height: the maximum perpendicular distance from the neck plane of the dome, Width: the maximum diameter perpendicular to H_max_, Neck width: the largest cross-sectional diameter of the aneurysm neck plane, aspect ratio (AR) equals to height/neck width, size ratio (SR) equals to H_max_/D_V_, and D_V_ denote the average of the diameter of the cross-sectional a vessel (Da) just proximal to the neck of the aneurysm and the diameter of the cross-section (D_b_) at 1.5 times Da from the neck of the aneurysm.

The morphological parameters of cerebral aneurysms mainly include the size, shape, location, and the ratio between various geometrical parameters, which indicates the crucial role in determining the risk of rupture (Kleinloog et al., 2018; Zhu et al., 2020; Baharoglu et al., 2010; Leemans et al., 2019; Liu et al., 2019; Tang et al., 2022). This review aims to provide a comprehensive overview of the relationship between morphological parameters and the risk of rupture in cerebral aneurysms, as shown in Figure 2.

**Size:** The size of the aneurysm is one of the most extensively studied morphological characteristics associated with rupture risk (Yi et al., 2016a; Merritt et al., 2021; Malhotra et al., 2017). Larger aneurysms are generally considered to have a higher risk of rupture, although the size threshold for high risk remains controversial. Some studies suggest that aneurysms larger than a certain size, such as 7 mm or 10 mm, have an increased risk of rupture, while others argue that smaller aneurysms can also be at risk (Kim H. C. et al., 2019; Xiong et al., 2022). Additionally, the rate of aneurysm expansion over time may serve as a significant determinant in assessing the likelihood of rupture.

**Shape:** The shape of an aneurysm is another important morphological characteristic that influences the risk of rupture (Kleinloog et al., 2018; Jirjees et al., 2020; Fung et al., 2019; Forget et al., 2001). Aneurysms with irregular shapes, such as multilocular or fusiform configurations, have been associated with an increased risk of rupture compared to those with a more spherical shape. The irregular shape may indicate areas of weakness in the aneurysm wall or increased wall stress, which can predispose the aneurysm to rupture.

**Location:** The location of an aneurysm within the cerebral vasculature also plays a significant role in determining the risk of rupture (Kleinloog et al., 2018; Can and Du, 2016; Park et al., 2018). Aneurysms located at the terminal ends of arteries or at bifurcations are more prone to rupture due to the increased hemodynamic forces and stress concentrations in these areas. Additionally, certain locations, such as the posterior communicating artery or the middle cerebral artery, have been identified as high-risk regions for rupture.

**Intraluminal Thrombus:** The presence of intraluminal thrombus within an aneurysm has been identified as a potential risk factor for rupture (Kleinloog et al., 2018; Wermer et al., 2007; Wang et al., 2019). Thrombus formation can alter the hemodynamics within the aneurysm and increase wall stress, potentially leading to rupture. The size and distribution of thrombus within the aneurysm may also influence the risk of rupture.

**Aspect Ratio (AR):** AR is the ratio of the perpendicular height of the aneurysm to the neck width of the aneurysm. Aneurysm with high ARs will have deep domes and small necks. An elevated AR is conducive to the recirculation of blood and the creation of low-velocity flow states, which have been detected in aneurysms that have ruptured. Such low-velocity conditions correlate with reduced WSS and an escalation in inflammatory responses within the endothelium. The expansion and rupture of aneurysms may take place in regions where wall shear stress is diminished, a situation that is probable in aneurysms characterized by a high AR and pronounced dome depths. AR of ≥1.5 is significantly increase the risk of rupture, which is regarded as strongest predictor of rupture status for aneurysms (Boussel et al., 2008; Carter et al., 2006; Sanchez et al., 2023; Dhar et al., 2008; Reneman et al., 2006).

**Size Ratio (SR):** Another key morphological parameter is the SR, defined as the maximum aneurysm height relative to the average parent vessel diameter (Kleinloog et al., 2018; Waqas et al., 2020; Jirjees et al., 2020; Fung et al., 2019). A high SR implies a larger aneurysm relative to the parent vessel and has been associated with increased wall tension and a greater likelihood of rupture. Aneurysms with high SRs have deep domes compared with the diameter of their parent vessels. SRs are significantly different between ruptured and unruptured aneurysms. It is previously reported that Studies have identified a SR ≥ 2.3 as a potential cutoff value for predicting aneurysm rupture (Sanchez et al., 2023). This threshold could potentially be employed to assess the rupture risk rather than only relying on the maximum diameter of the aneurysm, which could precisely predict the rupture of small aneurysms with the diameter <5 mm. However, it is important to note that while these values serve as useful indicators, the precise thresholds can vary based on the population studied and the specific methodology used in the study.

In the previous studies, the morphology of cerebral aneurysms, including size, shape, location, intraluminal thrombus, and size ratio, has played a critical role in determining the risk of rupture. However, the relationship between these morphological characteristics and rupture risk is complex and can vary among individuals. It is crucial to understand that while these measures provide valuable insights, they represent a snapshot of the aneurysm’s current state and do not fully encapsulate the dynamic nature of aneurysm growth and progression. Moreover, these morphological parameters are derived from three-dimensional structures projected onto two-dimensional images, which may not always accurately reflect the true aneurysm morphology. Therefore, while morphological parameters can help guide clinical decisions, they should be interpreted within the context of other patient-specific factors such as age, gender, and genetic predisposition.

Despite the relative simplicity and general applicability of these risk factors, they tend to overlook the intricacies of aneurysm behavior. While they provide a reasonable approximation of rupture risk, they fail to capture the complex, multifaceted nature of cerebral aneurysms, which may lead to inaccuracies in risk prediction. Currently, these traditional risk assessment methods have several limitations. Firstly, they often oversimplify the complexity of aneurysm behavior by focusing on static anatomical features, ignoring the dynamic nature of blood flow and its interaction with the vessel wall. Secondly, their predictive performance in clinical practice has shown to be inconsistent, with many small aneurysms rupturing while some large aneurysms remain stable. Finally, they do not account for individual patient variability in hemodynamics, vessel wall biology, and biomechanical properties, all of which could influence aneurysm growth and rupture.

This highlights the importance of pursuing a more comprehensive approach that factors in the intricate interplay of mechanical, biological, and hemodynamic elements in aneurysm formation, growth, and rupture. Advances in imaging techniques and computational models have improved our ability to assess morphological features and their implications for aneurysm rupture. Further research is needed to validate these findings and develop robust tools for accurate risk assessment and prediction of cerebral aneurysm rupture.

## Hemodynamical risk factors

**FIGURE 3:**
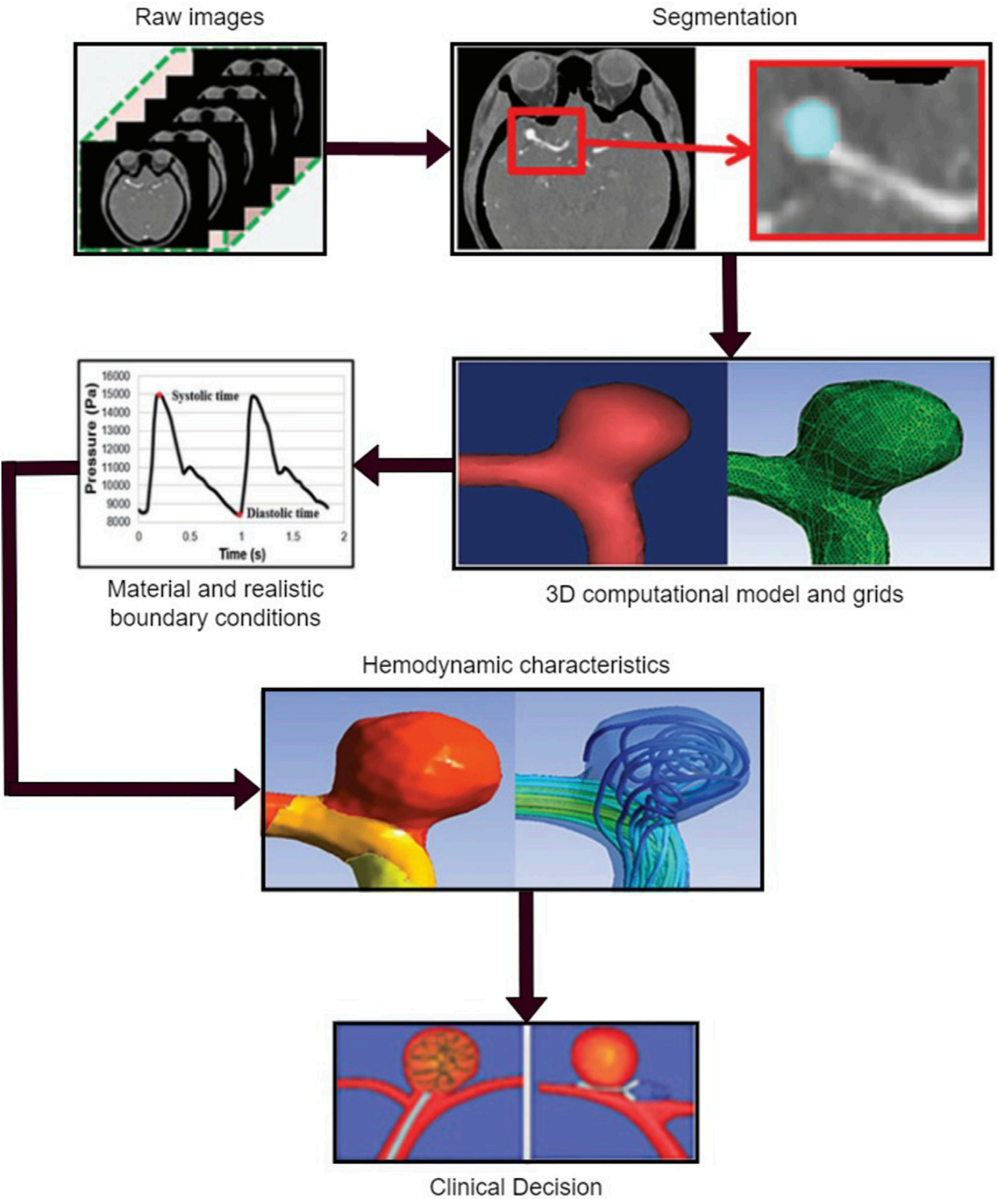
Hemodynamic analysis of a patient-specific aneurysmal model, including obtaining the raw data, medical segmentation concentrating on the aneurysm zone, three-dimensional (3D) reconstructed model for the computation and mesh the 3D model, define the material properties and load the realistic boundary conditions on the inlet and outlet of the computational model, obtain the flow characteristics related to the quantified hemodynamic parameters, and clinical decision for physicians.

Hemodynamics is a branch of physics that concerns the principles governing blood flow in the cardiovascular system, which is fundamental to understanding the pathophysiology of numerous cardiovascular conditions, including cerebral aneurysms. Hemodynamic forces, primarily including pressure, flow velocity, and WSS, are key determinants of vascular health and function, influencing everything from vascular tone and remodeling to endothelial function and inflammatory responses (Xiang et al., 2014; Baharoglu et al., 2010; Leemans et al., 2019; Cho, 2023). Hemodynamical computation procedure for the patient-specific model is as shown in Figure 3.

WSS is a particular hemodynamic parameter of great importance. It is a tangential force exerted by the flowing blood on the vascular wall (Staarmann et al., 2019; Zhang et al., 2016; Meng et al., 2014; Zhou et al., 2017). Normal WSS values are crucial for maintaining vascular integrity and endothelial health. However, abnormal WSS, either too high or too low, can result in endothelial damage and dysfunction, which can pave the way for aneurysm initiation and progression. High WSS, for instance, can directly damage the vascular endothelium, leading to endothelial denudation and promoting a pro-inflammatory state that can stimulate aneurysm growth. On the other hand, areas of low WSS are often characterized by disturbed flow, which can induce endothelial cell apoptosis, inflammatory cell adhesion, and extracellular matrix degradation, all of which contribute to aneurysm formation and rupture.

Another critical hemodynamic parameter is the Oscillatory Shear Index (OSI), a dimensionless measure that quantifies the changes in WSS direction (Meng et al., 2014; Jirjees et al., 2020; Can and Du, 2016; Yamaguchi et al., 2016; Yin et al., 2018; Yin et al., 2016; Huang et al., 2021; Huang et al., 2016; Huang et al., 2018). Higher OSI values indicate a more disturbed flow, and these regions are often associated with the initiation and growth of aneurysms. Elevated OSI values cause endothelial cells to switch to a synthetic phenotype, promoting inflammation, matrix remodeling, and weakening the vascular wall, thereby contributing to aneurysm pathogenesis.

In addition to WSS and OSI, pressure (Kleinloog et al., 2018; Penn et al., 2011) and flow velocity (Can and Du, 2016; Turjman et al., 2014) also play a crucial role. High intra-aneurysmal pressure can cause the aneurysm to expand and eventually rupture. Likewise, high flow velocity within the aneurysm can result in increased WSS and turbulent flow, contributing to aneurysm growth and rupture.

The interplay of these hemodynamic forces can be influenced by several factors, including aneurysm characteristics such as size, location, and morphology. For example, larger aneurysms often exhibit slower flow and lower WSS, creating an environment conducive to aneurysm progression. Similarly, aneurysms located at bifurcations or curvatures of the cerebral arteries, where blood flow is naturally disturbed, often experience abnormal hemodynamic forces, contributing to their development and progression. Aneurysms with a high AR (more spherical) or a high SR (large relative to the parent vessel) are likely to experience unstable and high-velocity flow, which can result in increased WSS, high OSI, and elevated intra-aneurysmal pressure, all of which heighten the risk of aneurysm rupture.

Overall, the hemodynamic forces and the way they interact with the vascular wall and aneurysm characteristics play a pivotal role in the pathogenesis and progression of cerebral aneurysms. A deeper understanding of these forces and their interplay could lead to better prediction models and more effective management strategies for patients with cerebral aneurysms.

## Pathology and mechanisms

To understand the reasons behind the rupture of some cerebral aneurysms, it is essential to conduct a meticulous study of their histopathological characteristics and molecular biology. Early histopathology studies through the classical histological staining techniques have confirmed that wall of cerebral aneurysms is characterized by lack of internal elastic lamina and normal intima-media-adventitia layers (Frösen et al., 2012; Hassler, 1961). These studies also showed the presence of inflammatory cells identified as polymorphonuclear leukocytes, plasma cells and small round cells (lymphocytes) (Chyatte et al., 1999; Stehbens, 1963).

From the observations of the histopathology using immunostaining techniques, it has been reported that the rupture of cerebral aneurysm is closely associated with the structural degeneration and infiltration of inflammatory cells in the wall (Frosen et al., 2004). Meanwhile, loss of endothelium, loss of mural cells, breaking down of the collagen matrix and partial hyalinization of the wall have been found in association with rupture of the wall (Kataoka et al., 1999).

Comparing the gene expression of ruptured and unruptured cerebral aneurysms with genome-wide microarrays have been investigated, which shows the overexpression of collagenases (matrix metalloproteinase 2 and 9), pro-apoptotic genes and inducible nitric oxide (NO) synthetase in ruptured cerebral aneurysms, as well as downregulation of anti-apoptotic genes. It is also found 1,426 differently regulated genes of signaling pathways associated with inflammatory cell infiltration, oxidative stress, disturbed cell homeostasis and dysfunctional endothelium were upregulated in ruptured cerebral aneurysms (Kurki et al., 2011).

Rupture of cerebral aneurysms occurs when the matrix of cerebral aneurysms wall has degenerated sufficiently fragile leading to resist the hemodynamic pressure of the cerebral arteries. Normally, collagen fibers would be repaired and maintained by a series of continuous synthesis of new collagen in case of being exposed to abnormally mechanical stress eventually leading to degenerate eventually (Sawabe and international, 2010; Bruno et al., 1998). Meanwhile, walls of ruptured cerebral aneurysm are distinguished by a significant depletion of mural cells (Frosen et al., 2004; Kataoka et al., 1999; Frösen et al., 2006; Kim et al., 1997). When mural cells are lost in the walls, the essential ‘repair and maintenance’ cycle, which relies on matrix synthesis and the proliferation of mural smooth muscle cells is impaired. The absence of mural cells, coupled with the continuous ‘wear and tear’ on collagen fibers and the ongoing proteolytic injury in some cerebral aneurysm walls, which could make the cerebral aneurysms more susceptible to rupture (Frösen et al., 2012). Consequently, the disappearance of mural cells is a pivotal factor contributing to the degeneration and ultimate rupture of cerebral aneurysm walls.

Intraluminal thrombosis, which is linked to the degeneration and rupture of cerebral aneurysms (Frosen et al., 2004), serves as a significant generator of oxidative stress. This stress arises from the activity of peroxidases released by neutrophils that become entangled within the thrombus (Houard et al., 2009). It is reported that oxidative stress may lead to mural cell death in the walls of cerebral aneurysms caused by the accumulation of cytotoxic oxidated lipids in the wall (Frösen et al., 2012). Besides, oxidative stress could indirectly induce inflammation by oxidative modification of intramural lipids of the wall, which would become immunogenic and trigger inflammatory response.

Hence, the advent of innovative techniques capable of detecting endothelial dysfunction, luminal thrombus formation, mural cell death, or inflammation holds promise for identifying cerebral aneurysms at high risk of rupture. These methods could be instrumental given the correlation between such findings and the presence of degenerate, rupture-prone walls in histopathological studies. Beyond enhancing the diagnostic capabilities for cerebral aneurysms prone to rupture, a profound understanding of the wall’s pathobiology may pave the way for the development of novel pharmaceutical or biological therapies. Such interventions could potentially tip the balance in favor of “repair and maintenance” over “cell death and wall degradation”, thereby which would reduce the ruptured risk of cerebral aneurysms instead of necessitating surgical or other invasive procedures to provide better therapeutic strategy for physicians.

## Hemodynamics and vascular mechanobiology

Abnormal hemodynamics act as a trigger for vascular remodeling, which indicates that remodeling of arteries as a biological response aimed at normalizing either increased or decreased WSS to a healthy range of 15–20 dynes/cm^2^ (Hashimoto et al., 2006; Lu and Kassab, 2011). Specifically, the biological processes of vascular remodeling contain vascular smooth muscle cells (VSMCs) apoptosis and migration, degradation of the extracellular matrix (ECM), and inflammation, which lead to local dilation and thinning the vessel wall (Hudson et al., 2013; Penn et al., 2014; Chien and Physiology, 2007; Vasconcellos et al., 2010). Furthermore, it is important to show that the biological response of vascular remodeling in the initiation of cerebral aneurysm is a distinct process from the growth and rupture of cerebral aneurysm.

Under conditions of hemodynamic stress, the release of NO in a flow-dependent manner can inhibit the proliferation of VSMCs and trigger apoptosis by activating the caspase 3 enzyme, which results in pyknosis, karyorrhexis, and ultimately cell death (Vasconcellos et al., 2010). Additionally, mechanical stretch of the vessel wall can induce VSMC apoptosis through the activation of the tumor suppressor protein p53, which is primarily located in the medial layer of blood vessels. VSMC migration is a typical response to vascular injury and is primarily directed towards the intima, the innermost layer of the blood vessel. This migration is influenced by various chemical signals such as amines, peptide growth factors, cytokines, and components of ECM, which contributes to the thinning of the vessel wall (Penn et al., 2014). Migration of VSMCs is conducted by the release of molecules like tumor necrosis factor-alpha (TNF-α) and Kruppel-like transcription factor 4, which lead to a change in the VSMC phenotype. Hence, VSMCs start to express genes associated with upregulation of pro-inflammatory molecules, including matrix metalloproteinases (MMPs), monocyte chemoattractant protein-1 (MCP-1), vascular cell adhesion molecule-1 (VCAM-1), and interleukin (IL) (Signorelli et al., 2015). VSMCs in the intimal layer can proliferate and synthesize new matrix and fibrous tissue, which is a process known as intimal hyperplasia. Typically, in aneurysms that are prone to rupture, areas of decellularization have been identified as part of the wound-healing process, which show loss of mural cells, breakdown of matrix, and hyalinization resembling fibrinoid necrosis (Frösen et al., 2012).

The disappearance of the internal elastic lamina is one of the first changes seen in the tissue structure during aneurysmal growth, leaving the adventitia as the only layer that can withstand the pressure of blood flow. The proteins in the ECM continue to deteriorate as MMPs break down structural proteins like collagens, elastin, proteoglycans, laminin, and fibronectin. Flow-dependent NO release also enhances MMP activity through posttranslational modification (Penn et al., 2014). Specifically, an overexpression of MMP-1, -2, and -9 has been observed in aneurysm walls, with higher levels of MMP-2 and -9 found in ruptured aneurysms (Kawaguchi et al., 2012). Chronic damage caused by collagenases is further increased by the downregulation of antiapoptotic genes (Frösen et al., 2012).

Monocytes are one of the initial cell types to respond to damage to the endothelium and infiltrate the injured site, where they differentiate into macrophages. These macrophages secrete cytokines and proteinases, including MCP-1, TNF-a, and stromal cell–derived factor-1(SDF-1, also known as CXCL12). SDF-1/CXCL12 helps recruit endothelial progenitor cells and promote angiogenesis, as well as further inflammatory cell migration and infiltration (Hoh et al., 2014). Additionally, macrophages contribute to ECM degradation by secreting MMPs. CD163-positive macrophages, the primary macrophages found in cerebral aneurysms, express a specific receptor and are triggered by high levels of oxidative stress contributing to cerebral aneurysm wall degeneration and rupture (Frösen et al., 2012; Fabriek et al., 2005; Lamping et al., 2000; Fisslthaler et al., 2000). Macrophage infiltration has also been linked to intracellular lipid accumulation and apolipoproteins, such as ApoA-I (Ollikainen et al., 2016) and ApoB100 (Frösen et al., 2013).

The difference in atherosclerotic lesions between small and large aneurysms is significantly evident. In small aneurysms, the lesions are characterized by diffuse intimal thickening with mainly VSMCs and minimal macrophages and lymphocytes. However, larger aneurysms have more advanced atherosclerotic lesions with macrophages being the primary cellular infiltrate (Kosierkiewicz et al., 1994).

Humoral immune response is active in the walls of aneurysms, with complement components and antibodies (Immunoglobulin M [IgM] and [IgG]) being deposited (Frösen et al., 2012). Accumulation of C3d has been found in aneurysms, which shows a potentially involved chronic inflammatory state. Activation of the classic pathway, along with amplification of the alternative pathway, has been implicated in releasing chemokines that recruit macrophages and T cells. Membrane attack complexes are rarely found on the cell surface, but are instead located within degraded regions, suggesting complement activation may be a reaction to necrosis rather than the cause of it (Lu and Kassab, 2011).

To understand the correlation between hemodynamics and vascular remodeling associated with cerebral aneurysm rupture is of profound significance in the field of neurovascular medicine, which could predict the risk of rupture, provide appropriate strategies, and potentially guide the development of targeted therapies to stabilize vulnerable aneurysms. Moreover, understanding the interplay between hemodynamics and vascular biology provides a foundation for personalized medicine approaches, where treatment can be tailored based on an individual’s unique aneurysm characteristics and hemodynamic profile, ultimately aiming to prevent rupture and improve patient outcomes.

## Imaging modality

**FIGURE 4:**
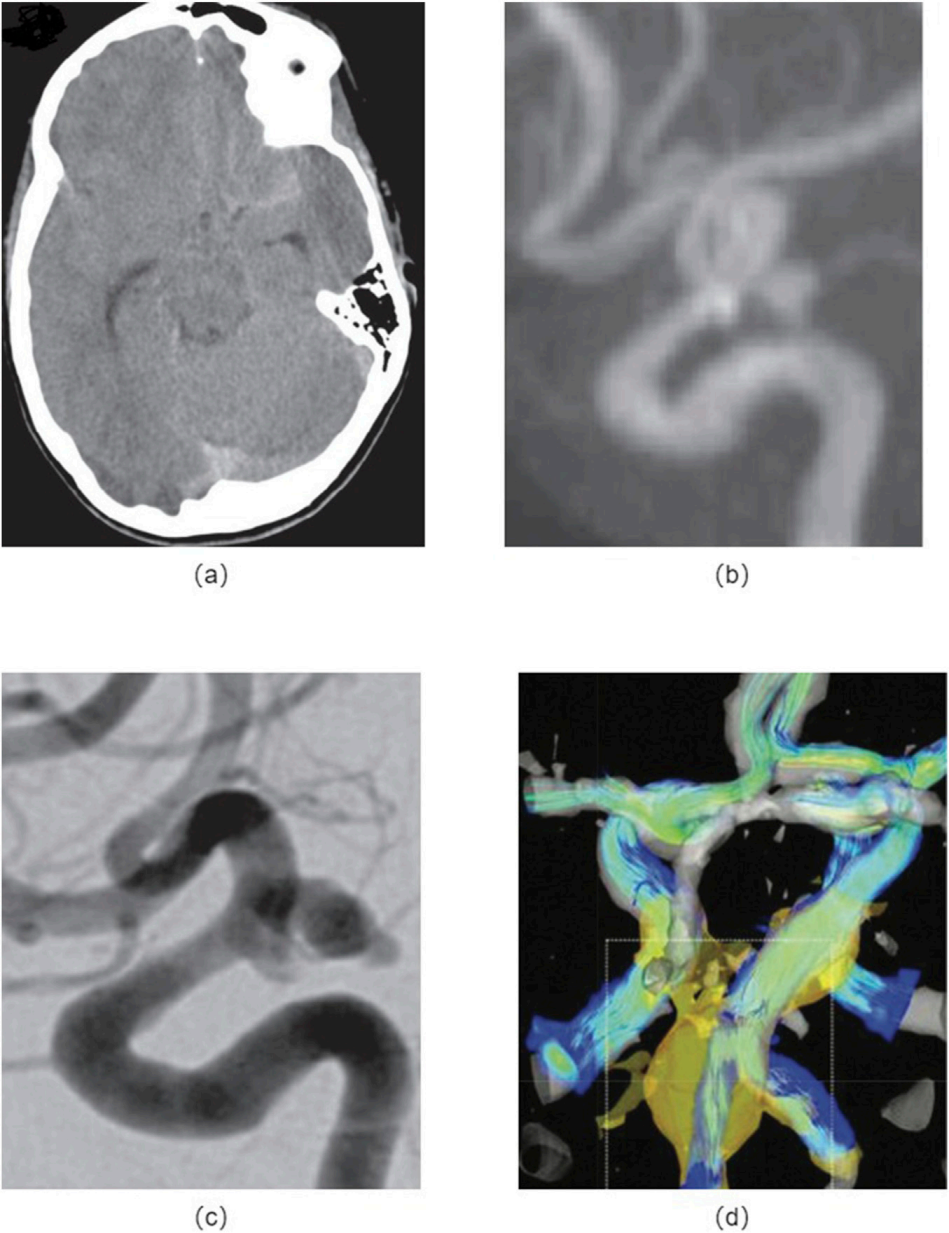
Medical imaging methods: **(A)** Non-contrast head CT images of a patient with the cerebral aneurysm; **(B)** MRA shows a left posterior communicating artery aneurysm; **(C)** DSA indicates an enlarged posterior communicating artery aneurysm; **(D)** 4D-MRI of the fusiform aneurysm shows the peripheral fast flow channel associated with high WSS.

Cerebral aneurysms are required to have early and accurate medical intervention immediately, which is crucial for effective treatment and management. In recently years, significant advancements have been made in imaging modalities, enabling healthcare professionals to diagnose cerebral aneurysms with higher accuracy and precision. Herein, there are routinely various imaging techniques used in the detection and diagnosis of cerebral aneurysms, as shown in Figure 4.

Computed Tomography Angiography (CTA) has been widely used for the detection of cerebral aneurysms (Diab et al., 2023; Yi et al., 2016a; Fung et al., 2019; Yi et al., 2016b; Villablanca et al., 2013; Shi et al., 2020a; Howard et al., 2019). It involves the injection of a contrast agent into the patient’s bloodstream, followed by a series of X-ray images. CTA provides detailed images of the blood vessels in the brain, allowing for the identification of aneurysms. However, it may have limitations in detecting smaller aneurysms or those located in complex anatomical regions.

Magnetic Resonance Angiography (MRA) utilizes magnetic fields and radio waves to create detailed images of the blood vessels in the brain (Diab et al., 2023; Yi et al., 2016a; Howard et al., 2019; van Amerongen et al., 2014; Ahmed et al., 2019). It is a non-invasive procedure and does not involve the use of ionizing radiation. MRA can provide valuable information about the size, location, and characteristics of cerebral aneurysms. However, it may have limitations in visualizing smaller aneurysms or those in challenging anatomical locations.

Digital Subtraction Angiography (DSA) is considered the gold standard for diagnosing cerebral aneurysms (Fung et al., 2019; Howard et al., 2019; van Amerongen et al., 2014; Ahmed et al., 2019). It involves the injection of a contrast agent into the blood vessels, followed by X-ray imaging. DSA provides highly detailed and accurate images of the cerebral vasculature, allowing for precise detection and characterization of aneurysms. However, it is an invasive procedure and carries a slightly higher risk compared to non-invasive imaging techniques.

4D-Flow Magnetic Resonance Imaging (4D-MRI) is an advanced imaging technique that provides a comprehensive evaluation of blood flow dynamics in the brain (Castle-Kirszbaum et al., 2020; Szajer and Ho-Shon, 2018; Pereira et al., 2016; Turski et al., 2016). It can accurately measure flow velocities, directions, and volumes within the cerebral arteries, allowing for the detection of aneurysms and assessment of their hemodynamic characteristics. This technique is particularly useful in evaluating the risk of rupture and planning treatment strategies.

In clinical practice, various imaging modalities above have been conducted to obtain the medical images for the patients with cerebral aneurysms by the radiologist, it should be precisely and deliberately chosen with the appropriate imaging technique based on the individual patients’ clinical presentation and anatomical characteristics to ensure accurate diagnosis and effective treatment planning (Hesamian et al., 2019).

## Blood biomarkers

**FIGURE 5:**
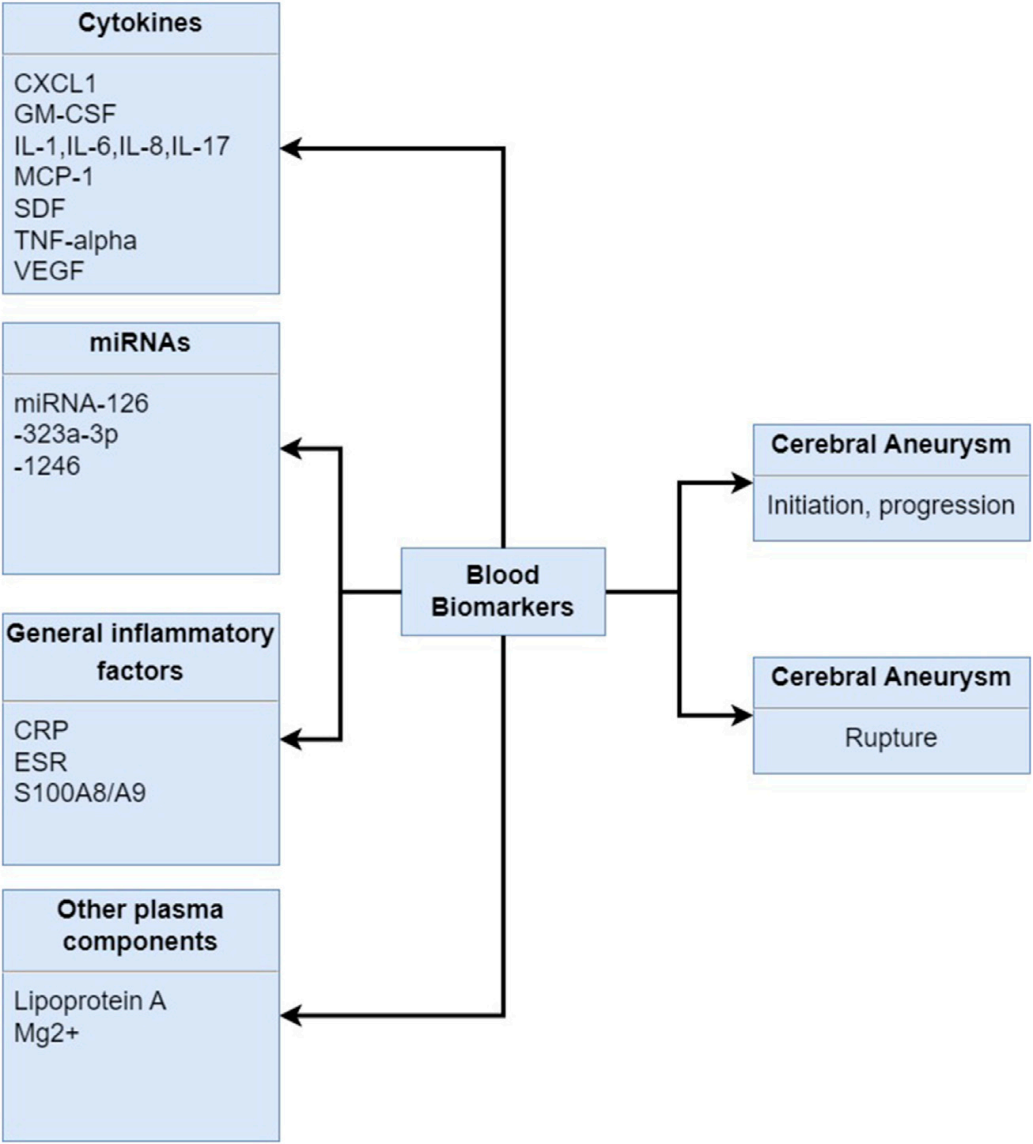
Blood biomarkers in human samples associated with the initiation, progression and rupture of the cerebral aneurysms. CXCL1: chemokine C-X-C motif ligand 1, GM-CSF: granulocyte-monocyte colony-stimulating factor, IL-1: interleukin-1, IL-6: interleukin-6, IL-8: interleukin-8, IL-17: interleukin-17, MCP-1: monocyte chemoattractant protein-1, SDF: stromal cell-derived factor, TNF-alpha: tumor necrosis factor--alpha, VEGF: vascular endothelial growth factor, CRP: C-reactive protein, ESR: erythrocyte sedimentation rate, S100A9/A9: S100 family of proteins.

Early detection and monitoring of cerebral aneurysms are crucial for timely intervention and management performed by the imaging modalities, which have been the primary method for diagnosing cerebral aneurysms. Meanwhile, recent research has focused on the identification of blood biomarkers that could complement imaging techniques (Hussain et al., 2015; Jordan and Nyquist, 2010; Nowicki et al., 2023; Wu et al., 2023). Herein, there are distinct findings of blood biomarkers in the diagnosis, prognosis, and risk assessment of cerebral aneurysms, as shown in Figure 5.

**Inflammatory and Immunological Markers:** Inflammation and immune response play critical roles in the pathogenesis and progression of cerebral aneurysms. Studies have identified various inflammatory and immunological markers, such as C-reactive protein (CRP) (Wu et al., 2023; Al-Mufti et al., 2017; Przybycien-Szymanska et al., 2015), interleukin-6 (IL-6) (Wu et al., 2023; Jabbarli et al., 2020; Simon and Grote, 2021; Monsour et al., 2023; Watson et al., 2018), and tumor necrosis factor-alpha (TNF-α) (Hussain et al., 2015; Wu et al., 2023; Watson et al., 2018; Tsilimigras et al., 2018), as potential indicators of aneurysm instability and rupture risk. Elevated levels of these markers in the blood have been associated with aneurysm growth and increased risk of subarachnoid hemorrhage.

**Extracellular Matrix Biomarkers:** The extracellular matrix (ECM) provides structural support to the vessel walls and is subject to remodeling processes in response to hemodynamic forces and inflammation. Biomarkers of ECM remodeling, such as matrix metalloproteinases (MMPs) (Zhang et al., 2019; Li et al., 2017; Lattanzi et al., 2020), particularly MMP-2 (Mehta et al., 2013; Gareev et al., 2021) and MMP-9 (Zhang et al., 2019; Kassam et al., 2004), and tissue inhibitors of metalloproteinases (TIMPs) (Signorelli et al., 2018; Urbonavicius et al., 2008), have been implicated in aneurysm growth and rupture. Assessing the balance between ECM degradation and synthesis may offer insights into aneurysm stability and inform risk stratification.

**Endothelial Dysfunction and Oxidative Stress Biomarkers:** Endothelial dysfunction and increased oxidative stress contribute to vascular damage and play a role in aneurysm development (Signorelli et al., 2018). Biomarkers of endothelial dysfunction, such as von Willebrand factor (vWF) (Hussain et al., 2015; Ma et al., 2021) and asymmetric dimethylarginine (ADMA) (Siasos et al., 2015; Miao and Liao, 2014), and markers of oxidative stress (Signorelli et al., 2018; Hussain et al., 2015), such as malondialdehyde (MDA) (Husseini and Laskowitz, 2010; Rodríguez-Rodríguez et al., 2014) and 8-hydroxy-2′-deoxyguanosine (8-OHdG) (Duan et al., 2016; Korkmaz et al., 2018), have been explored as potential indicators of aneurysm risk. These markers reflect the vascular response to hemodynamic and inflammatory insults and may aid in risk assessment.

**Genetic and Epigenetic Biomarkers:** Advancements in genetic and epigenetic research have uncovered genetic variants and epigenetic modifications associated with aneurysm susceptibility and progression (Bakker and Ruigrok, 2021; Fernández-Pérez et al., 2024). Polymorphisms in genes involved in vascular biology, such as ACTA2 (Balmforth et al., 2019; Boileau et al., 2018), MYH11 (Wang et al., 2023; Saratzis and Bown, 2014), and COL3A1 (Kassam et al., 2004; Laurent et al., 2022), have been linked to aneurysm formation and rupture. Additionally, epigenetic changes, such as DNA methylation (Fernández-Pérez et al., 2024; Samuel and Radovanovic, 2019) and histone modifications (Fernández-Pérez et al., 2024), may regulate gene expression and contribute to aneurysm pathogenesis. These genetic and epigenetic factors hold promise as novel biomarkers for risk assessment and personalized medicine (Alg et al., 2013).

**Integration of Biomarkers:** The combination of multiple biomarkers may improve the diagnostic and prognostic accuracy of cerebral aneurysms (Kleinloog et al., 2018; Hussain et al., 2015). Research has suggested that a panel of biomarkers, including inflammatory, extracellular matrix, endothelial dysfunction, and genetic markers, could provide a more comprehensive assessment of aneurysm risk and aid in clinical decision-making. This approach may pave the way for personalized medicine, tailored monitoring and treatment strategies for patients with cerebral aneurysms.

Blood biomarkers offer a promising approach to complement existing imaging techniques in the detection, prognosis, and risk assessment of cerebral aneurysms. While individual biomarkers have shown potential, the integration of multiple biomarkers may enhance the diagnostic accuracy and predictive value. Future research should focus on validating these biomarkers in larger clinical studies and developing biomarker panels that can be effectively translated into clinical practice. The incorporation of blood biomarkers into routine clinical assessments could lead to more personalized and precise management strategies for patients with cerebral aneurysms.

## Artificial intelligence

**FIGURE 6:**
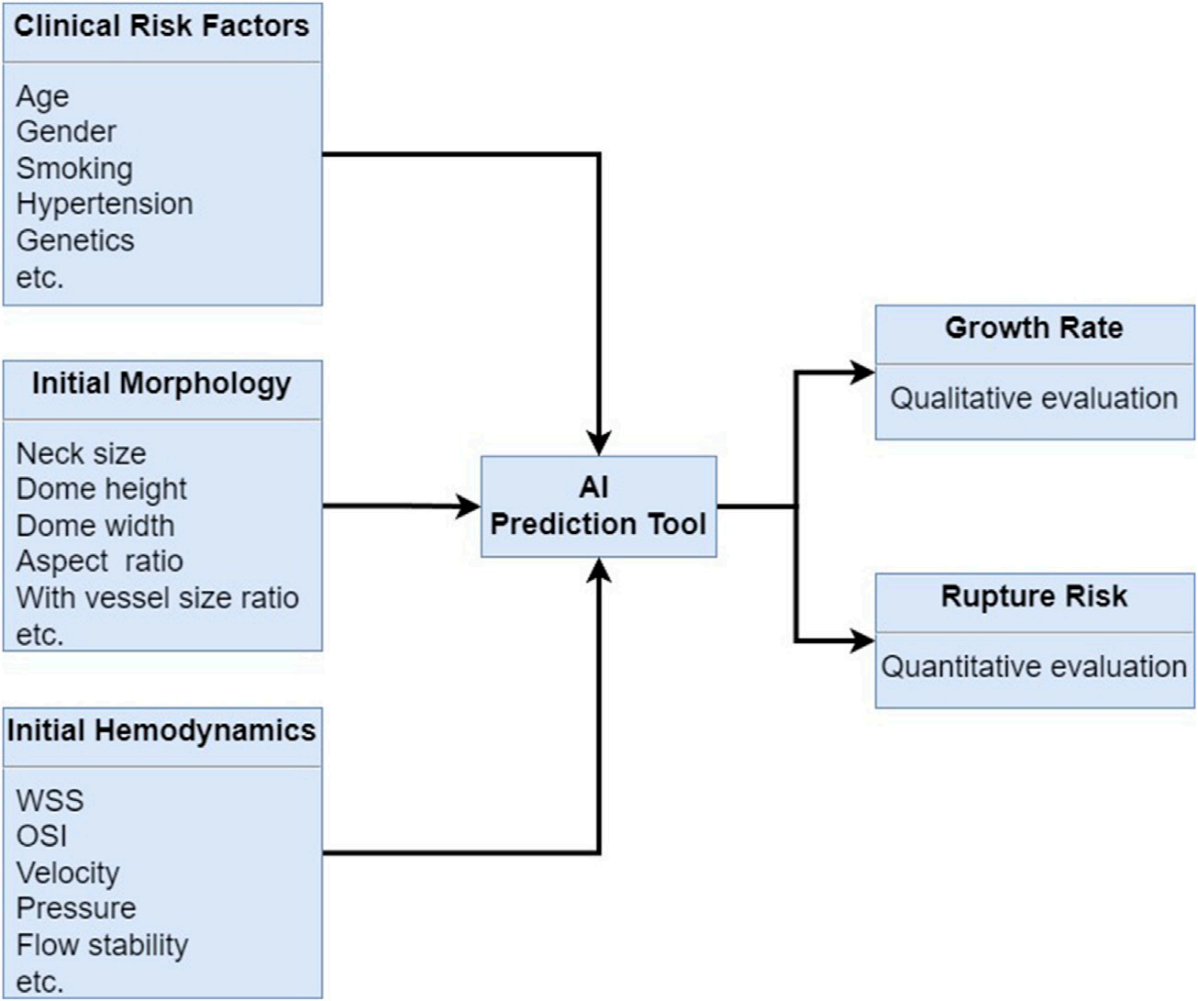
AI algorithm prediction for cerebral growth rate and rupture risk by combining the clinical risk factors, initial morphology and initial hemodynamics.

Recent advancements in artificial intelligence (AI) have shown great promise in enhancing the detection and management of these aneurysms (Yang et al., 2021; Zhu et al., 2020; Liu et al., 2019; Xiong et al., 2022; Larson et al., 2018; Mouridsen et al., 2020; Jin et al., 2020; Ahn et al., 2021; Detmer et al., 2020; Salman et al., 2023; Lin et al., 2022). AI has emerged as a powerful tool for enhancing the detection, risk assessment, and treatment planning of cerebral aneurysms. AI algorithms have shown great potential in improving the accuracy and efficiency of diagnosis, as well as providing valuable insights into aneurysm prognosis and treatment outcomes (Kim D. W. et al., 2019; Kim H. C. et al., 2019; Shi et al., 2020a; Hesamian et al., 2019; He et al., 2019; Wang and Summers, 2012; Liu et al., 2018; Heo et al., 2020; Nabaei, 2022; Din et al., 2023; Marasini et al., 2022). Herein, the role of AI in improving the diagnosis, risk assessment, and treatment planning for cerebral aneurysms are discussed in the following study, as shown in Figure 6.

**AI in Cerebral Aneurysm Detection:** AI algorithms, particularly deep learning convolutional neural networks (CNNs), have demonstrated remarkable accuracy in detecting cerebral aneurysms from CTA and MRA images (Hosny et al., 2018). These algorithms can automatically identify and segment aneurysms, reducing the workload on radiologists and improving the efficiency of diagnosis. Several studies have shown that AI-based detection systems can achieve high sensitivity and specificity, often matching or surpassing the performance of human experts.

**AI in Risk Assessment and Prognosis:** AI algorithms can analyze various imaging features and clinical data to assess the risk of aneurysm rupture and predict patient outcomes. By integrating information such as aneurysm size, shape, location, hemodynamics, and blood biomarker related to the rupture of cerebral aneurysm, AI systems can provide valuable insights into the likelihood of aneurysm growth and rupture. This information can help clinicians in making informed decisions regarding the need for surgical intervention and the choice of treatment strategy.

**AI in Treatment Planning:** AI can also play a significant role in treatment planning for cerebral aneurysms. For instance, AI algorithms can assist in simulating blood flow dynamics within the cerebral vasculature, helping to identify regions at high risk of rupture. Additionally, AI can aid in the selection of treatment modalities, such as endovascular coiling or surgical clipping, by predicting the success rates and potential complications associated with each approach.

Despite the promising results, several challenges need to be addressed for the widespread adoption of AI in cerebral aneurysm management (Chen et al., 2022; Hesamian et al., 2019; Recht et al., 2020; Shi et al., 2020b). These include the need for large, diverse, and annotated datasets for training AI algorithms, ensuring the generalizability of these models across different populations and imaging protocols, and addressing issues related to data privacy and security. Future research should focus on developing robust AI systems that can be seamlessly integrated into routine clinical practice, as well as exploring the potential of AI in personalized medicine for cerebral aneurysm patients. Overcoming the current challenges and continued advancements in AI technology are expected to further revolutionize the field of cerebral aneurysm management, ultimately leading to better patient care and outcomes (Jiang et al., 2017).

## Fluid-structure interaction

**FIGURE 7:**
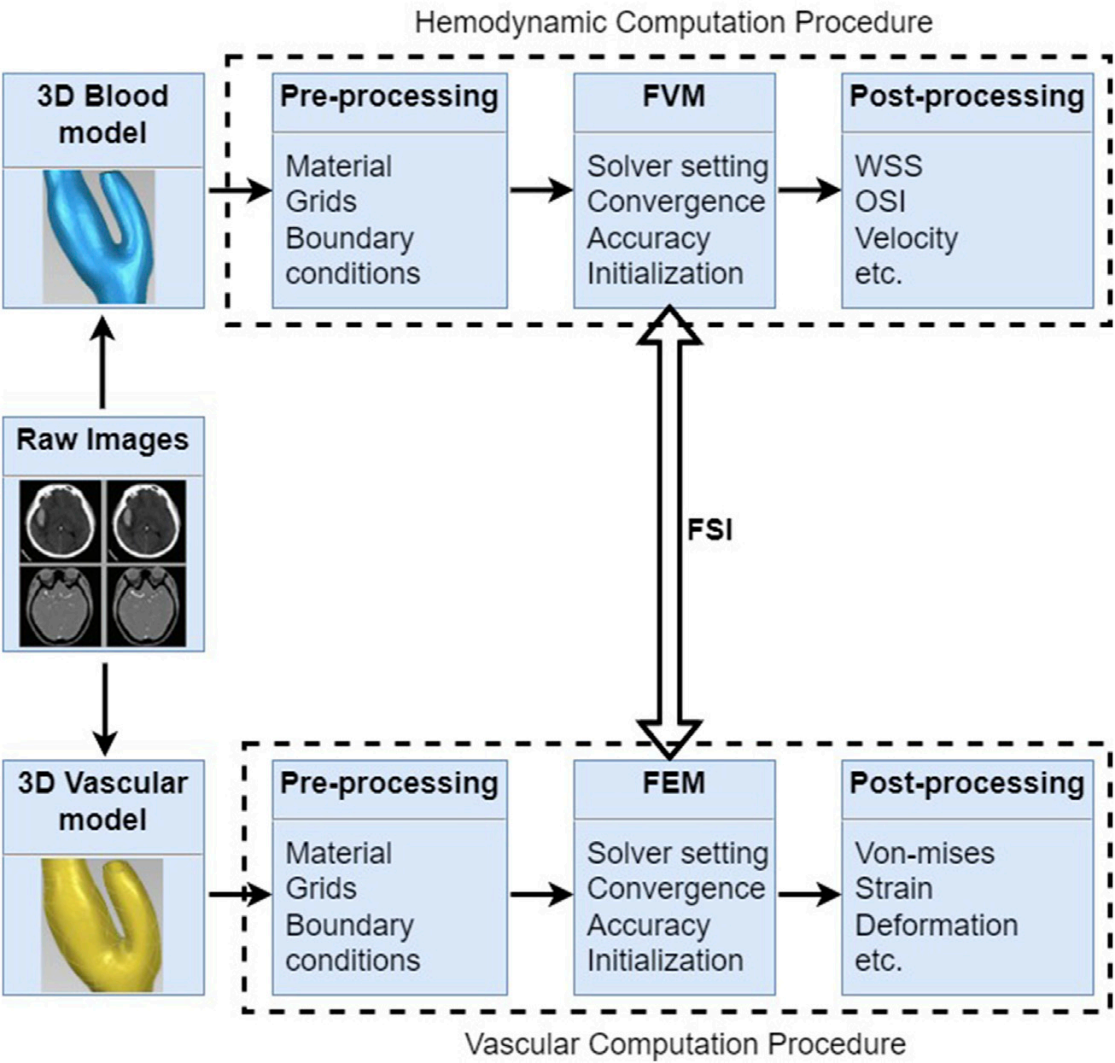
Fluid-structure interaction procedures for cerebral aneurysms include hemodynamic computation and vascular computation, which shows 3D reconstructed model, pre-processing, computational methods and post-processing, respectively.

Fluid-structure interaction (FSI) analysis is a computational modeling technique that simulates the dynamic interaction between blood flow and the vessel walls, providing valuable insights into the hemodynamics and structural stresses involved in cerebral aneurysms (Kleinstreuer et al., 2007; Nazri et al., 2020; Turjman et al., 2014; Mourato et al., 2022; Bai-Nan et al., 2011). Understanding the mechanics behind aneurysm development, growth, and rupture is essential for improving diagnosis, risk assessment, and treatment strategies in clinical practice. FSI could simulate the relatively physiology conditions for patients, which considers the interaction between the vessel and blood, as shown in Figure 7.

FSI computation allows for the detailed examination of blood flow patterns and hemodynamics within cerebral aneurysms. Studies have shown that certain flow characteristics, such as high WSS, flow impingement (Can and Du, 2016), and high velocity jets, can contribute to aneurysm initiation and growth. By simulating these flow dynamics, FSI analysis can help identify regions at high risk of aneurysm formation and rupture, guiding clinical decision-making regarding treatment options.

The structural response of the aneurysm wall to hemodynamics is a critical factor in determining its stability and risk of rupture. FSI computation enables the evaluation of wall stress and deformation patterns, providing insights into the mechanical environment that predisposes aneurysms to rupture. By incorporating material properties of the vessel wall and studying the effects of wall remodeling, FSI analysis can enhance our understanding of the mechanical mechanisms underlying aneurysm growth and rupture.

FSI computation holds great promise for personalized medicine in the context of cerebral aneurysms. By simulating patient-specific geometries and hemodynamic conditions, FSI models can provide tailored information on the risk of aneurysm rupture and assist in treatment planning. For instance, FSI simulations can predict the distribution of WSS after endovascular coiling or surgical clipping, aiding in the selection of the most appropriate treatment strategy for individual patients.

While FSI computation has made significant contributions to our understanding of cerebral aneurysmal hemodynamics, several challenges remain to be addressed. These include the development of accurate and validated constitutive models for the vessel wall, the integration of biological factors such as inflammation and remodeling processes, and the requirement of larger-scale clinical studies to validate the predictive capabilities of FSI models. In the further study, FSI computation combined the previouslly realsitic challenges should be considered and applicable in routine clinical practice, which will promote the personalized medicine, treatment planning, and ultimately improving patient outcomes in the management of cerebral aneurysms.

## Limitations and challanges

Although various risk factors have been proposed in predicting the rupture of cerebral aneurysms in the previous studies, these methods have inherent limitations and challenges that affect the predictive accuracy and clinical utility. Herein, there are limitations and challenges associated with the risk assessment of cerebral aneurysms in the previous study.

**Morphological Characteristics:** Morphological features, such as aneurysm size, shape, location, and aspect ratio, have been used to assess the risk of rupture. However, several limitations exist: (1) Size alone is an imperfect predictor, as small aneurysms can rupture, while larger ones may remain stable; (2) The complexity of aneurysm shapes makes it challenging to quantify risk, and subjective assessments can lead to variability among interpreters; (3) The relationship between aneurysm geometry and rupture risk is not fully understood, and the impact of geometric changes over time remains unclear.

**Hemodynamical Characteristics:** Hemodynamic factors, such as blood flow patterns, WSS, and pressure gradients, are crucial in aneurysm pathogenesis. However, their use in risk assessment has challenges: (1) The correlation between specific hemodynamic patterns and rupture risk is not always consistent, and the threshold values for high-risk conditions are not well-defined; (2) Hemodynamic simulations require accurate aneurysm geometry and boundary conditions, which can be difficult to obtain, especially in the presence of intraluminal thrombus or altered vascular wall properties; (3) The invasive nature of methods like phase-contrast magnetic resonance imaging (MRI) or measurement of intravascular pressure limits their applicability in routine clinical practice.

**Intrasubject Variability:** There is considerable intrasubject variability in aneurysm characteristics, which can change over time due to factors such as inflammation, remodeling, and changes in blood flow. This variability makes it challenging to establish a baseline for risk assessment and necessitates repeated evaluations, which may not be practical or cost-effective.

**Lack of Standardization:** The lack of standardized definitions and criteria for aneurysm risk assessment tools contributes to variability in interpretation and communication between clinicians and researchers. This hinders the development of consensus guidelines and the validation of risk assessment models across different studies and centers.

**Integration of Multimodal Data:** While morphological and hemodynamical characteristics are important, they represent only part of the complex picture of aneurysm risk. Integrating these data with other clinical and biological factors remains a challenge, and the potential of multimodal data fusion for improved risk assessment is yet to be fully realized.

In summary, advances in imaging technology, computational modeling, and data analysis techniques are required to address these issues. Standardization of definitions and criteria, development of robust validation frameworks, and integration of multimodal data are key steps toward improving the accuracy and clinical utility of risk assessment tools for cerebral aneurysms.

## Conclusion

Rupture risk factors and predictors in cerebral aneurysms were performed through various methods related to clinical perspective, morphological parameters, hemodynamic parameters, pathology and mechanisms, vascular mechanobiology, imaging modalities, blood biomarkers, artificial intelligence, and fluid-structure interaction, which showed important significance in diagnosis, progression and patient-specific treatment. Meanwhile, current limitations and challenges of the previous methods to predict the rupture of cerebral aneurysms were outlined and should be enhanced to identify in the further study. By comprehending and pinpointing the diverse risk factors and predictive biomarkers linked to aneurysm rupture, we can enhance personalized risk evaluation and refine therapeutic approaches for individuals with cerebral aneurysms.
